# A contemporary review of breast cancer risk factors and the role of artificial intelligence

**DOI:** 10.3389/fonc.2024.1356014

**Published:** 2024-04-18

**Authors:** Orietta Nicolis, Denisse De Los Angeles, Carla Taramasco

**Affiliations:** ^1^ Engineering Faculty, Universidad Andres Bello, Viña del Mar, Chile; ^2^ Centro para la Prevención y Control del Cáncer (CECAN), Santiago, Chile

**Keywords:** breast cancer, risk factors, artificial intelligence (AI), medical history, metabolic factors, reproductive and hormonal factors, lifestyle factors, environmental influence

## Abstract

**Background:**

Breast cancer continues to be a significant global health issue, necessitating advancements in prevention and early detection strategies. This review aims to assess and synthesize research conducted from 2020 to the present, focusing on breast cancer risk factors, including genetic, lifestyle, and environmental aspects, as well as the innovative role of artificial intelligence (AI) in prediction and diagnostics.

**Methods:**

A comprehensive literature search, covering studies from 2020 to the present, was conducted to evaluate the diversity of breast cancer risk factors and the latest advances in Artificial Intelligence (AI) in this field. The review prioritized high-quality peer-reviewed research articles and meta-analyses.

**Results:**

Our analysis reveals a complex interplay of genetic, lifestyle, and environmental risk factors for breast cancer, with significant variability across different populations. Furthermore, AI has emerged as a promising tool in enhancing the accuracy of breast cancer risk prediction and the personalization of prevention strategies.

**Conclusion:**

The review highlights the necessity for personalized breast cancer prevention and detection approaches that account for individual risk factor profiles. It underscores the potential of AI to revolutionize these strategies, offering clear recommendations for future research directions and clinical practice improvements.

## 1 Introduction

Over the past decade, breast cancer has remained a leading cause of mortality among women globally, driving an intensive search for effective prevention and early detection strategies. During 2020, more than 2.3 million women were diagnosed, of which 33.5% died (1). Despite significant advances in understanding biological mechanisms and risk factors of breast cancer, substantial challenges persist in the personalized clinical management and preventive intervention. This work aims to evaluate and synthesize the evidence available on breast cancer risk factors, ranging from genetic predispositions and lifestyle to environmental influences, with a particular interest in recent technological advancements, including AI, in predicting and detecting the disease. We pose two critical research questions: 1) What are the main risk factors associated with the development of breast cancer, and how do these vary among different populations and age groups? 2) How do recent technological advancements based on Artificial Intelligence (AI) help the detection and prevention of breast cancer? Guided by the hypothesis that the variability in breast cancer risk factors among different populations suggests that prevention and early detection strategies must be personalized, considering genetic, lifestyle, and environmental factors to be effective, this review seeks to identify areas of consensus and discrepancy in the scientific literature. Highlighting the need for personalized strategies that consider variability among populations and age groups, we aim to provide clear recommendations that guide future research and clinical practices towards more effective prevention and early detection of breast cancer.

The paper is organized as follows. In Section 2, the methodology for selecting and reviewing papers is described. Section 3 shows the results with particularly emphasis to the bibliometric study and risk factor categories. A discussion and some conclusions are in Sections 5 and 6, respectively.

## 2 Methodology

**Figure 1:**
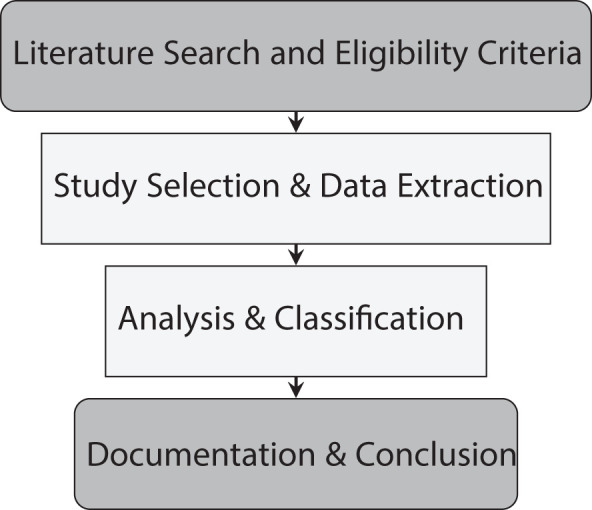
Flow chart of the methodology.

The methodology of the paper involved a comprehensive bibliographic development and analysis, which steps are described in **Figure 1**.

### 2.1 Literature search and eligibility criteria

Our review concentrated on studies published between 2020 and 2024, with a focus on breast cancer risk factors. We sourced these from databases like PubMed, Scopus, and Web of Science. We included research papers that provided insights into demographic, genetic, lifestyle, and environmental influences on breast cancer risk, alongside studies utilizing AI for enhancing risk prediction and classification. Exclusion criteria were set for articles published prior to 2020 and those not directly examining the outlined risk factors. English language has been mainly used for the selection.

### 2.2 Study selection and data extraction

The study selection process meticulously filtered approximately 250 article by titles, abstracts and keywords, to determine their relevance to breast cancer risk factors and AI applications. A deeper process based on a complete reading of the papers narrowed the focus to 112 articles that met our inclusion criteria and offered important information on the topic. This approach ensured that only the most relevant studies were included, providing a detailed exploration of breast cancer risk factors and the role of AI in risk management. A bibliometric analysis was realized for setting frequencies and relationships among risk factors. Finally, these risk factors were systematically classified into categories, as detailed in **Table 1**.

**Table 1:** Keywords and descriptions for breast cancer risk factors and AI research.

Risk Factor	Keywords and search
Demographic and Genetic Factors	
Age	*Breast cancer age risk, age-related breast cancer*
Race or ethnicity and geographic location	*Breast cancer ethnicity, racial disparities in breast cancer, geographic variation breast cancer*
Family History	*Family history breast cancer risk, hereditary breast cancer*
Genetic mutations	*HER2 (Human Epidermal Growth Factor Receptor 2), Genetic and Molecular Factors*
Economic factors	*Socioeconomic status breast cancer risk, Socioeconomic impact breast cancer, economic disparities breast cancer*
Reproductive and Hormonal Factors	
Menarche (age at first menstruation)	*Menarche breast cancer risk, hormonal exposure breast cancer*
Menopause (age at menopause)	*Menopause breast cancer risk, hormonal exposure breast cancer*
Breastfeeding and Parity (number of fullterm pregnancies)	*Breastfeeding breast cancer risk reduction, parity breast cancer correlation*
Hormonal factors (use of hormone replacement therapy, contraceptives, etc	*HRT (hormone replacement therapy) breast cancer risk, contraceptives breast cancer*
Metabolic Factors	
Diabetes	*Insulin resistance breast cancer, glycemic control breast cancer, type 2 diabetes*
Metabolism	*Thyroid function breast cancer*,
Medical History	
Breast density	*Breast density cancer risk, mammographic density breast cancer*
Other cancers and diseases	*Comorbidity breast cancer risk, second primary cancer breast cancer*
Lifestyle Factors	
Alcohol consumption	*Alcohol consumption breast cancer risk*
Cigarette smoking	*Smoking and breast cancer link*
Obesity and Body Mass Index (BMI)	*obesity breast cancer correlation, dietary factors affecting breast cancer*
Poor nutrition	*Dietary fats and breast cancer, Nutritional deficiencies and breast cancer*
Physical inactivity	*Exercise breast cancer risk reduction, physical inactivity breast cancer*
Stress, anxiety, or depression	*Stress and breast cancer risk, depression impact on breast cancer, anxiety breast cancer correlation*
Environmental Factors	
Exposure to radiation	*Radiation exposure breast cancer risk, ionizing radiation breast cancer*
Exposure to chemicals	*Endocrine disruptors breast cancer risk, chemical exposure and breast cancer*
Environmental pollutants and heavy metals	*Explore research on how air quality and exposure to pollutants correlate with breast cancer incidence*, *Research the relationship between exposure to industrial byproducts and breast cancer development*
Use of AI in Risk Prediction	
*AI breast cancer detection, predictive models Breast Cancer.*	

### 2.3 Analysis and classification

This classification was based on the analysis of risk factors available in various articles, which were then grouped according to characteristics to derive the respective classifications. Regarding risk factors, they were classified into groups corresponding to “Demographic and Genetic Factors”, “Reproductive and Hormonal Factors”, “Metabolic Factors”, “Medical History” and “Lifestyle and Environmental Factors.” Additionally, a new independent category was created to group papers that include studies with artificial intelligence models, named “Use of AI in Risk Prediction”. A simple Natural Language Processing (NLP) word count was used to identify the risk factors most frequently mentioned in each paper.

### 2.4 Documentation and conclusion

This methodology involved the following steps: conducting an exhaustive literature search across major scientific databases; applying inclusion and exclusion criteria, and to narrow down the selection from approximately 250 papers to 112 most relevant papers; employing techniques for a more deep analysis of the risk factors mentioned across the selected papers and categorizing the identified risk factors into specific groups for a structured analysis. This methodology not only ensures a comprehensive understanding of the existing research landscape but also supports the identification of key risk factors for breast cancer, facilitating a more precise and evidence-based analysis.

## 3 Results

By applying the above methodology, we show the results of the a systematic literature review of the selected 112 papers and we describe the main findings for each category of risk according to **Table 2**.

**Table 2:** Summary of risk factors and characteristics in breast cancer research literature.

Paper	Age	Race and geography	Family History	Genetic mutations	Economic factors	Menarche	Menopause	Breastfeeding and Parity	Hormonal factors	Diabetes	Metabolism	Breast density	Other cancers and diseases	Alcohol consumption	Cigarette smoking	Obesity and BMI	Poor nutrition	Physical inactivity	Stress, anxiety, or depression	Exposure to radiation	Exposure to chemicals	Pollutants and heavy metals	AI Models
(2)		•	•		•																		
(3)				•					•	•						•							
(4)		•		•					•														
(5)																							•
(6)																						•	
(7)																							•
(8)																							•
(9)		•									•												
(10)				•					•														
(11)	•											•											
(12)		•		•																			
(13)			•	•							•												
(14)																							•
(15)	•																						
(16)																	•						
(17)																							•
(18)		•		•																			
(19)							•									•		•					
(20)							•									•							
(21)				•			•		•								•						
(22)																•				•			
(23)								•	•														
(24)	•			•																			
(25)							•		•							•							
(26)						•	•	•	•							•							
(27)											•					•							
(28)																							•
(29)		•					•									•							
(30)		•					•		•		•												
(31)									•														
(32)		•			•			•															
(33)	•	•						•				•				•							
(34)										•													
(35)	•		•	•										•		•							
(36)					•																		
(37)				•					•														
(38)	•							•										•		•			
(39)		•																					
(40)														•									
(41)															•								
(42)	•	•										•											
(43)									•														
(44)																	•						
(45)																							•
(46)	•											•											
(47)		•																					
(48)																							•
(49)		•				•	•				•						•	•					
(50)																							•
(51)					•				•					•									
(52)									•														
(53)	•			•			•																
(54)				•																			
(55)			•	•																			
(56)				•																			•
(57)																			•				
(58)																				•			
(59)				•					•														
(60)																							•
(61)		•							•														
(62)																	•						
(63)																							•
(64)	•							•															
(65)	•							•															
(66)		•											•										
(67)	•	•														•							
(68)							•				•					•							
(69)	•		•									•				•							
(70)					•																		
(71)				•			•									•							
(72)						•	•	•	•														
(73)																							•
(74)																							•
(75)											•												
(76)							•		•							•							
(77)			•	•																			
(78)									•														
(79)																•							
(80)		•						•						•	•								
(81)																							•
(82)				•																			
(83)	•												•										
(84)																							•
(85)														•									
(86)					•																		
(87)																•	•						
(88)																							•
(89)		•		•																			
(90)	•	•														•							
(91)			•	•								•						•					
(92)				•																			
(93)																•	•						
(94)																•	•						
(95)																							•
(96)							•															•	
(97)			•	•																			
(98)				•																•			
(99)				•					•														
(100)		•																					
(101)		•	•	•																			
(102)							•									•							
(103)																	•	•	•				
(104)		•	•	•																			
(105)																					•		
(106)													•							•			
(107)													•										
(108)												•											
(109)											•												
(110)	•		•																				
(111)							•		•											•			
(112)		•																					
(113)											•												

### 3.1 Bibliometric analysis

In this section we provide a bibliometric analysis using the Bibliometrix package of R software (114).

**Figure 2:**
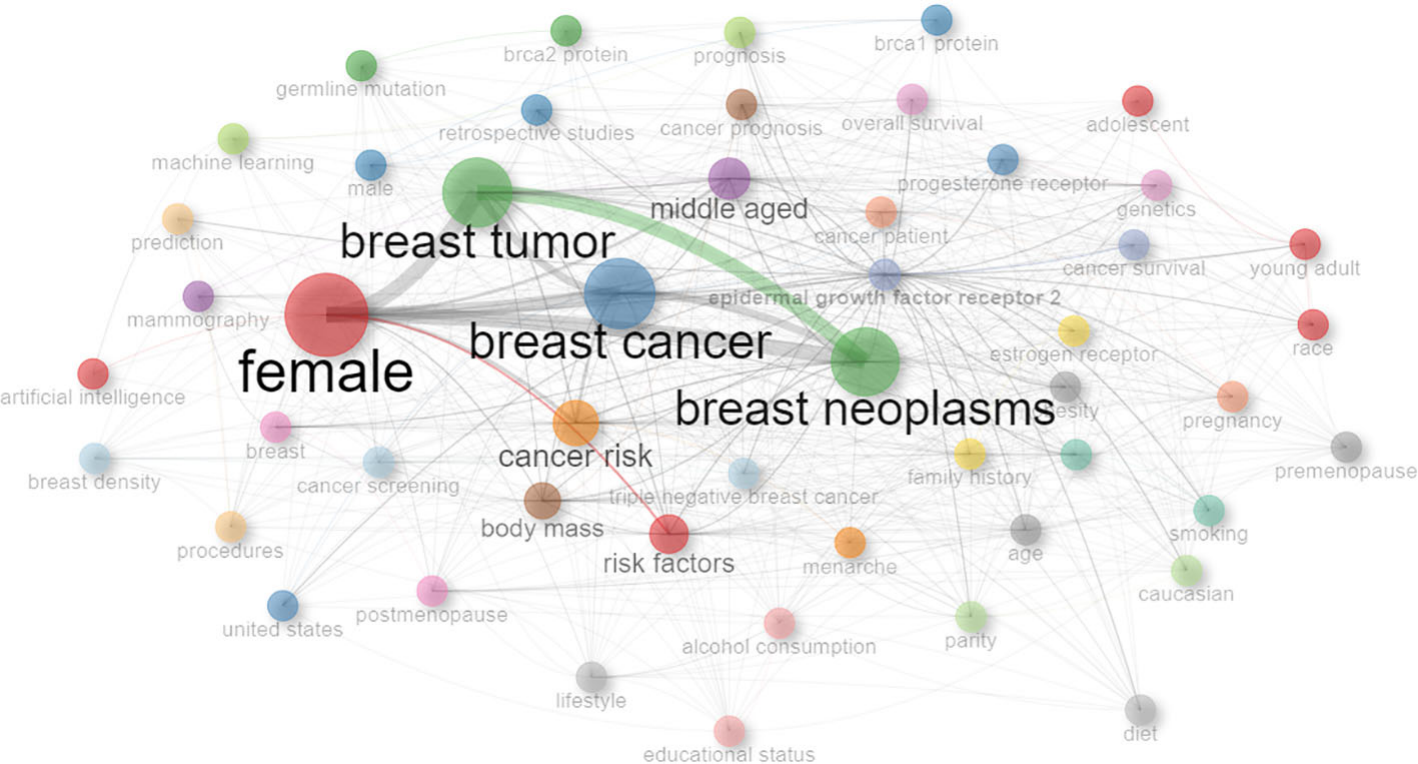
Keyword network visualization in breast cancer research.

In order to facilitate a deeper understanding of how keywords interconnect across the collection of reviewed papers, a keyword network graph is shown in **Figure 2**. The graph highlights the thematic ties and focal points within the research landscape under examination. In the **Figure 2** we can see the most interconnected and frequent keywords are: female, breast tumor, breast cancer and breast neoplasms.

**Figure 3:**
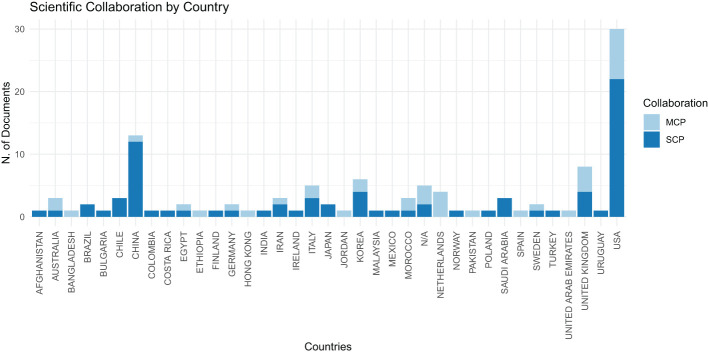
Distribution of countries of bibliographic authors.

**Figure 3** displays the distribution of bibliographic authors by country. In this chart, ‘MCP’ represents Multiple Country Publications, indicating research papers co-authored by individuals from various nations, while ‘SCP’ signifies Single Country Publications, denoting research executed solely by authors within the same country. This visual representation clearly indicates that the United States is at the forefront in terms of the volume of scientific publications, with significant contributions in both national (SCP) and international (MCP) collaborations, followed by China, evidencing a robust level of scientific output and cooperative engagement in these nations.

**Figure 4:**
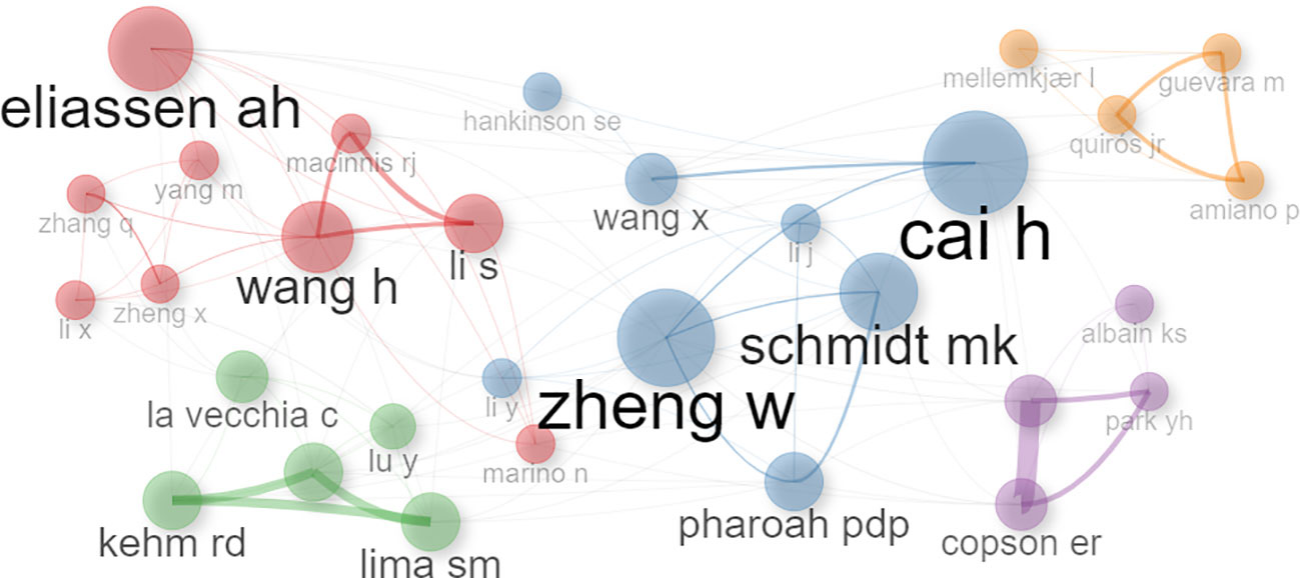
Co-authorship network analysis in scientific research.

Conversely, the author network depicted in **Figure 4** illustrates clustering among authors who have contributed to more than five publications. Those with a higher publication frequency are represented by larger circles, visually highlighting the most prolific contributors within the network.

### 3.2 Breast cancer risk factors

In this Section, we provide a detailed analysis of breast cancer risk factors identified by the reviewed works as represented in **Table 2**.

### 3.3 Demographic and genetic factors

- Age: Age plays a crucial role in breast cancer incidence and outcomes, particularly impacting middle-aged and older women. Studies like (53) and (33) investigate treatment efficacy and risk factors, especially in younger women. Demographic factors, including age, are highlighted by (67) and (110). Mortality rates, notably rising in women under 50 and over 70, are observed by (65), underscoring age’s significance. Associations between reproductive history and breast cancer subtypes in women aged ≤50 are explored by (24) (42). focuses on mammographic density’s relation to risk in women aged 40 to 74. Lastly (46), emphasizes age-specific preventive measures for women aged 30–39.
- Race or ethnicity and geographic location: Research underscores significant variations in breast cancer predisposition across ethnicities and geographic locations, influenced by genetic, environmental, and socioeconomic factors. Studies like (112) emphasize diverse risk prediction models’ necessity, especially for Asian women. Disparities persist despite similar treatments, as shown by (4) among Black and White women. Meanwhile (12), and (18) identify genetic susceptibility in Egyptian and Arab populations. Geographical variations, highlighted by (29), highlight the need to adopt personalized approaches. These findings emphasize the multifaceted nature of breast cancer risk and treatment strategies across diverse populations.
- Family History: The presence of a family history significantly impacts the assessment and management of breast cancer risk (110). reveals that 35.5% of women with a familial history face a high lifetime risk, yet only 23.9% receive enhanced screening (13). demonstrates the effectiveness of machine learning, achieving 77.78% precision in risk prediction. In addition (77), identifies specific germline variants linked to susceptibility. Furthermore, the integration of polygenic risk scores with family history, as demonstrated by (91), significantly alters surveillance recommendations. Overall, these findings underscore the crucial role of family history in personalized breast cancer care and risk management.
- Genetic mutations, such as BRCA1 (Breast Cancer Gene 1) and BRCA2 (Breast Cancer Gene 2): Genetic mutations, particularly in BRCA1 and BRCA2 genes, significantly increase hereditary breast cancer risk. Studies like (92) analyze the role of germline CHEK2 (Checkpoint Kinase 2) variants, while (97) advocate personalized prevention strategies (98). identifies genetic loci associated with contralateral breast cancer risk, and (3) explores molecular links between obesity and breast cancer. These findings emphasize the multifactorial nature of breast cancer, requiring tailored risk assessment and management.
- Economic factors: Economic factors significantly impact breast cancer risk and outcomes (86). reveals disparities in access to systemic anticancer therapies based on geographic and sociodemographic factors. Similarly (36), notes a social gradient in cancer incidence in Costa Rica (51). links higher education levels to increased breast cancer risk (2). emphasizes local demographic factors in TNBC (Triple-Negative Breast Cancer) treatment, while (32) highlights access disparities in Colombia. Finally (70), stresses the importance of socio-demographic indices and public health policies in addressing breast cancer burden in developing countries.

### 3.4 Reproductive and hormonal factors

- Menarche (age at first menstruation): Early menarche increases breast cancer risk due to prolonged hormonal exposure (26). links higher anti-Müllerian hormone levels to early menarche, indicating elevated risk. Conversely (72), suggests later menarche protects against certain breast cancer subtypes. Lifestyle changes, like plant-based diets, are crucial in mitigating risk, as emphasized by (49).
- Menopause (age at menopause): Late menopause increases breast cancer risk due to prolonged hormonal exposure (111). links menopausal hormonal changes to chemotherapy side effects severity. Conversely (20), emphasizes fat distribution’s role in postmenopausal breast cancer risk (26). associates lower anti-Müllerian hormone levels with earlier menopause, indicating elevated risk. Conversely (72), suggests later menopause as a risk factor for certain breast cancer subtypes. Lifestyle factors like higher BMI and caloric intake heighten post-menopausal breast cancer risks, as noted by (49).
- Breastfeeding and Parity (number of full-term pregnancies): Parity and breastfeeding reduce breast cancer risk (80). analyzes parity’s influence across birth cohorts, showing changing risk patterns (26). links anti-Müllerian hormone levels to age at menarche and parity, aiding risk assessment (64). studies parity’s impact on breast cancer incidence, highlighting rising rates in younger women (72). meta-analysis reveals subtype-specific risks, emphasizing tailored prevention strategies.
- Hormonal factors (use of hormone replacement therapy, contraceptives, etc.): Hormonal factors like hormone replacement therapy and contraceptives influence breast cancer risk (3). highlights obesity’s role in breast cancer, especially in postmenopausal women (10). emphasizes hormonal imbalances’ impact, urging further research (59). finds no significant difference in breast cancer risk with Hormone Replacement Therapy among BRCA mutation carriers. These findings emphasize the importance of hormonal markers like estrogen and progesterone receptors in breast cancer treatment (3, 10, 59). Additionally (21), and (72) explore lifestyle factors like diet and reproductive behaviors, highlighting hormonal influences on breast cancer risk.

### 3.5 Metabolic factors

- Diabetes: Elevated levels of insulin can promote cellular proliferation and reduce apoptosis, thus facilitating the development and progression of mammary neoplasms (3). elucidate obesity’s pivotal role in breast cancer (BC) risk, particularly postmenopausal women, citing hormonal imbalances and insulin resistance among its mechanisms. They reveal how obesity-driven molecular changes, like increased estrogen and insulin levels, contribute to BC via specific signaling pathways. Conversely (34), find a significant correlation between genetic predisposition to Type 2 Diabetes Mellitus (T2DM) and poorer breast cancer-specific survival (HR = 1.10, 95% CI = 1.04–1.18, P = 0.003), emphasizing the potential causal impact of T2DM on BC outcomes.
- Metabolism: Metabolic processes play a crucial role in modulating breast cancer risk, significantly influencing hormonal levels and cellular dynamics. Alterations in metabolism, including imbalances in lipid and glucose metabolism, can lead to endocrine changes and alterations in the cellular microenvironment that favor mammary carcinogenesis. Metabolism plays a crucial role in breast cancer risk, with various factors influencing susceptibility (113). found that high-density lipoprotein cholesterol (HDL-C) significantly affects breast cancer risk, suggesting a metabolic component to cancer development (9). identified associations between insulin-like growth factor 1 (IGF-1) levels and fasting blood glucose with breast cancer risk, emphasizing the complexity of metabolic factors. Additionally (13), integrated genetic mutations and demographic factors to predict breast cancer risk, highlighting the importance of considering metabolic pathways in risk assessment. These findings underscore the multifaceted nature of metabolism-related risk factors in breast cancer susceptibility (113) (9) and (13).

### 3.6 Medical history

- Breast density: Breast density complicates cancer detection in the sense that it can make more difficult for mammograms to identify cancerous tumors due to the tissue’s thickness or opaqueness. Additionally, high breast density is considered an independent risk factor for developing breast cancer. This is because denser breast tissue contains more connective and glandular tissues, which can potentially hide tumors and it is also associated with a higher likelihood of cancer development (11). found a sixfold risk difference between densest and least dense categories (42). investigated this relationship across a cohort of 21,150 women, confirming the effectiveness of automated density assessments in predicting breast cancer risk. Similarly (69) emphasizes higher risk in younger women with lower BMI (46). explores mammography-based risk assessment for early screening. These studies underscore the importance of considering mammographic density in breast cancer risk assessment and screening.
- Other cancers and diseases: The presence of other cancers may indicate heightened risk for breast cancer (107). developed prognostic nomograms for breast cancer patients with lung metastasis (66). addressed disparities in colorectal and breast cancer screenings (83). revealed screening rate disparities among females with schizophrenia (106). noted a slight increase in primary lung cancer risk post-radiotherapy for breast cancer.

### 3.7 Lifestyle factors

- Alcohol consumption: Alcohol consumption significantly increases breast cancer risk, even with moderate intake (85). revealed odds ratios between 1.82 to 5.67, indicating a notable association (40). highlighted a high prevalence (18.34%) of risky drinking among Australian women, exceeding weekly guidelines. These studies emphasize the importance of preventive measures. These findings underscore the link between alcohol intake and breast cancer risk, highlighting the need for preventive measures (35, 51).
- Cigarette smoking: Cigarette smoking contributes to breast cancer risk, with global estimates from (41) showing it accounted for 5.1% of deaths and 5.2% of DALYs in 2019. They emphasize anti-tobacco policies, particularly in low SDI regions (80). found smoking’s heightened impact in younger Asian cohorts, highlighting the need for tailored prevention strategies.
- Obesity and Body Mass Index (BMI): Obesity, particularly postmenopause, significantly increases breast cancer risk, impacting hormonal levels and inflammation. Studies like (3) highlight obesity’s role in altering molecular pathways, while (102) emphasize its association with higher estrogen levels, especially in postmenopausal women (19). stresses lifestyle interventions for reducing breast cancer risk in obese postmenopausal women. Additionally (71), found BMI significantly influences breast cancer prognosis, particularly in premenopausal women with specific cancer subtypes.
- Poor nutrition: Poor nutrition, characterized by diets high in fats and sugars, increases breast cancer risk. Studies like (103) highlight the positive impact of tailored lifestyle interventions, while (16) suggest higher plasma vitamin D levels may offer protection (21). and (49) emphasize the association between Western diets and increased risk, contrasting with the protective effect of plant-based diets. Additionally (62), and (94) address dietary misconceptions and socio-demographic factors influencing nutritional risk, advocating for comprehensive approaches in breast cancer care.
- Physical inactivity: Physical inactivity increases breast cancer risk, while exercise helps regulate hormones and maintain a healthy weight. Studies like (19) emphasize its benefits in reducing recurrence risk. Tailored interventions, as shown by (103), positively impact survivors’ quality of life (49). link low physical activity to higher risk, especially in post-menopausal women. Additionally (91), propose personalized surveillance integrating lifestyle factors for better outcomes.
- Stress, anxiety, or depression: Chronic stress may impact breast cancer risk (57). links stress, anxiety, and depression to reduced quality of life in survivors (103). shows positive outcomes in QoL (Quality of Life) indicators with home-based interventions despite pandemic challenges.

### 3.8 Environmental factors

- Exposure to radiation: Exposure to ionizing radiation, like from radiotherapy, elevates breast cancer risk, especially when received at a young age. Studies explore various factors (38): concluded that exposure to chest radiation therapy significantly elevates breast cancer risk, with individuals who have undergone such treatments facing a notably higher likelihood of developing the disease. Similarly (57), mention that receiving chest radiation therapy was significantly associated with a higher risk of breast cancer, with an Adjusted Odds Ratio (AOR) of 6.43, indicating a more than sixfold increase in risk compared to those who had not received such therapy (98). found that genetic variations can influence an individual’s susceptibility to radiation toxicity (106). discusses lung cancer risk post-radiotherapy (111); links menopause to chemotherapy side effects; and (22) reported a high radiodermatitis incidence (98.2%) in breast cancer patients undergoing radiotherapy, with BMI and statin use affecting severity, and hydrogel showing protective effects.
- Exposure to chemicals: Chemicals like endocrine disruptors may disrupt hormonal balance, potentially contributing to breast cancer (105). evaluates CDK4/6 inhibitors’ toxicity in metastatic breast cancer, stressing personalized treatment strategies due to varying drug profiles.
- Environmental pollutants, specific exposures and heavy metals: Environmental pollutants, including heavy metals and air pollution, contribute to breast cancer risk (6). found altered levels of metals like copper and cadmium in breast cancer patients (96). investigated air pollution’s association with postmenopausal breast cancer risk, finding a significant 18% risk increase with a 10 µg/m3 rise in PM10 levels in 2007.

## 4 The role of artificial intelligence models for detecting breast cancer

The integration of artificial intelligence (AI) in breast cancer management spans various aspects, including diagnosis, recurrence prediction, survival rate estimation, and treatment response assessment. Studies like (5) demonstrate the effectiveness of machine learning models, achieving 80.23% accuracy in diagnosing early-stage breast cancer. Key risk factors identified for breast cancer included levels of glucose, age, and resistin. This approach demonstrates the potential of machine learning in enhancing breast cancer diagnostic processes by effectively selecting critical risk factors. Similarly (8), utilizes NLP and machine learning to predict breast cancer recurrence, emphasizing the efficacy of the OneR algorithm. The main clinical data used in the paper for predicting breast cancer recurrence involve a wide range of factors extracted from electronic health records (EHR). These include diagnostic symptoms, medications, lab results, medical recommendations, past medical history, procedures, family history, imaging, endoscopic assessments, anesthesia types, allergies, and other clinical documents. NLP algorithms were developed to extract these key features from the medical records. Notably (81), highlights Support Vector Machine (SVM) as the most accurate algorithm for breast cancer prediction, achieving an accuracy of 97.2%. The characteristics of the cell nuclei present in the images, are used as inputs for the SVM. They include, Radius, Texture, Area, Perimeter, Smoothness, Compactness, Concavity, Concave points, Symmetry, and Fractal dimension. These attributes are determined from the digitized images and serve as the basis for the SVM model to classify instances into benign or malignant categories.

For detection purposes, most of the papers use mammography images for training deep learning models, by assuming these algorithms are able to detect anomalies in the breast tissue. In this context, a comprehensive review is provided by (14) focusing on various ANN models such as Spiking Neural Network (SNN), Deep Belief Network (DBN), Convolutional Neural Network (CNN), Multilayer Neural Network (MLNN), Stacked Autoencoders (SAE), and Stacked De-noising Autoencoders (SDAE). The review highlights the effectiveness of these models in improving diagnosis accuracy, precision, recall, and other metrics, with particular success noted in models like ResNet-50 and ResNet-101 within the CNN algorithm framework. Instead, clinical data have been considered by (17) which developed a Machine Learning (ML) system for classifying breast cancer and diagnosing cancer metastases using clinical data extracted from Electronic Medical Records (EMRs). The best results have been obtained by a decision tree classifier which achieved 83% accuracy and an AUC (Area Under the Curve) of 0.87, demonstrating the potential of ML models based on blood profile data to aid professionals in identifying high-risk metastases breast cancer patients, thereby improving survival outcomes.

Regarding treatment response assessment (28), employs CNNs to predict treatment response in breast cancer patients undergoing chemotherapy, achieving high accuracies for various parameters. The study integrates both imaging and non-imaging data for the inputs of the models included longitudinal multiparametric MRI data (dynamic-contrast-enhanced MRI and T2-weighted MRI), demographics, and molecular subtypes. The use of advanced imaging techniques alongside clinical and molecular data indicates the need for a personalized treatment planning and assessment in breast cancer care (73). demonstrates deep learning’s superior performance in risk identification compared to traditional Machine Learning (ML) methods. Important inputs for their models include age, resistin levels, global burden of disease (GBD) relative risk upper values, glucose, adiponectin, high BMI (binary), MCP-1, leptin, relative risks from meta-analyses, obesity (binary), and insulin levels. These inputs were selected based on their relevance and low redundancy for predicting breast cancer, highlighting the potential of deep learning to complement traditional screening methods by identifying individuals at risk non-invasively and affordably. In survival rate prediction (63), evaluates ML’s role, highlighting challenges like data preprocessing and model validation. review 31 studies, mainly from Asia, to predict the 5-year survival rate of breast cancer. It is highlighted that among the papers reviewed, the most used algorithms are decision trees (61.3%), artificial neural networks (58.1%) and support vector machines (51.6%), where clinical and molecular information was used to build predictive models (73). used a database of 116 women, of which 52 were healthy and 64 had been diagnosed with breast cancer. The information included demographic and anthropometric data. The application of Deep Learning was considered the best evaluated method for breast cancer prediction, among algorithms such as SVM, Neural Networks, Logistic Regression, XGBoost, Random Forest, Naive Bayes and Stochastic Gradient. Lastly, studies like (88) predict patient satisfaction post-mastectomy, revealing that 45.2% of women experienced improved satisfaction with their breasts. These findings underscore the potential of AI in enhancing various aspects of breast cancer management, from diagnosis to patient satisfaction assessment. A novel approach that integrates Machine Learning (ML) algorithms with Explainable Artificial Intelligence (XAI) has been recently developed to enhance the understanding and interpretation of predictions made by ML models. In the context of breast cancer research (95), introduced a Hybrid Algorithm combining ML and XAI techniques aimed at preventing breast cancer. This innovative methodology enables the identification and extraction of key risk factors, such as high-fat diets and breastfeeding habits, to accurately differentiate between patients with and without breast cancer among Indonesian women. Risk indicators, such as auxiliary nodes and breast density, can also be extracted by the images by using deep learning (7, 56, 84).

## 5 Discussion

Upon reviewing multiple studies on breast cancer and its associated risk factors, several key findings emerge.

- Demographic and genetic factors play a crucial role in influencing breast cancer risk. This review highlights the crucial impact of age, with a notable increase in breast cancer incidence and outcomes, particularly affecting middle-aged and older women, as well as younger demographics in certain contexts. The significance of race, ethnicity, and geographic location is underscored, emphasizing the variability in breast cancer predisposition across different populations due to a mix of genetic, environmental, and socioeconomic factors. Family history and specific genetic mutations, such as BRCA1 and BRCA2, are identified as key risk determinants, necessitating personalized prevention and management strategies. Economic factors also emerge as crucial, with disparities in access to care and outcomes spotlighted. Collectively, these findings underscore the necessity for tailored breast cancer prevention and treatment approaches that consider the intricate interplay of demographic and genetic factors.
- Early menarche, late menopause, parity, breastfeeding, and hormonal therapies like hormone replacement therapy and contraceptives highly influence breast cancer risk. These factors are intricately linked with hormonal exposure over a woman’s lifetime, affecting her breast cancer susceptibility. This review emphasizes the need for awareness and consideration of these factors in breast cancer risk assessment, suggesting lifestyle modifications and preventive strategies tailored to individual reproductive histories and hormonal exposure profiles.
- The relationship between metabolic factors, such as diabetes and overall metabolism, play an important role in the context of breast cancer risk. In particular, conditions like insulin resistance and alterations in lipid and glucose metabolism can influence breast cancer development by affecting hormonal levels and cellular processes. Our review suggests that understanding the impact of these metabolic factors is crucial for developing targeted prevention strategies and emphasizes the need for further research to explore the intricate connections between metabolic health and breast cancer risk.
- Medical history, specifically breast density and the history of other cancers, can influence breast cancer risk. In particular, dense breast tissue can obscure mammograms, making detection more challenging, and emphasizes the independent risk factor that high breast density presents. Additionally, the history of other cancers may indicate an elevated risk for breast cancer. This work underscores the importance of considering an individual’s medical history in breast cancer risk assessments and the need for personalized screening strategies.
- Lifestyle factors such as alcohol consumption, cigarette smoking, obesity, poor nutrition, and physical inactivity, highlight their significant roles in increasing breast cancer risk and the necessity of addressing these modifiable risk factors through public health interventions and individual lifestyle changes to reduce breast cancer incidence. This review underscores the potential of preventive measures and lifestyle modifications in mitigating breast cancer risk, emphasizing the importance of holistic approaches in breast cancer prevention strategies.
- Environmental factors like radiation exposure, chemicals, and pollutants, play a significant role in breast cancer risk. The cited works emphasize the need for awareness and protective measures against these exposures. Highlighting the complexity of breast cancer etiology, our work calls for comprehensive research to better understand the interactions between environmental factors and genetic predisposition, and for public health strategies to minimize exposure and mitigate breast cancer risk.
- The description of role of artificial intelligence (AI) models in detecting breast cancer illustrates the significant potential AI has in enhancing diagnostic accuracy, predicting recurrence, estimating survival rates, and assessing treatment response. Highlighting various studies, this review shows that machine learning algorithms, such as Support Vector Machines (SVM) and Convolutional Neural Networks (CNNs), have achieved notable success. This discussion emphasizes AI’s transformative impact on breast cancer management, advocating for further research and integration of AI technologies to tailor detection and treatment approaches, ultimately improving patient outcomes.

A detailed description of the results of each work will be presented in Section 3.2. This analysis advocates for a multifaceted approach to prevention, screening, and treatment, reflecting the complex nature of breast cancer risk factors.

## 6 Conclusion

Our research reveals a breakthrough in early detection of breast cancer with machine learning models demonstrating an impressive diagnostic accuracy of 80.23%. The bibliographic review and analysis of the last 5 years in this field allowed us to identify the transformative impact of AI both in the identification of risk factors and in the improvement of diagnostic accuracy. Our analysis, unlike previous studies such as those by (69) (89), and (35), goes beyond updating risk factor inventories to show the fundamental role of sophisticated risk algorithms. AI. These tools, particularly SVM, have achieved an accuracy rate of up to 97.2% in locating breast cancer, which is a significant leap over traditional diagnostic methods by using a wider range of datasets, including images and clinical details including risk factors for your diagnosis.

Future explorations should delve into AI’s ability to tailor breast cancer detection and treatments, thereby improving patient-specific outcomes.

## Author contributions

ON: Writing – review & editing, Writing – original draft, Supervision, Methodology, Investigation, Conceptualization. DA: Writing – original draft, Software, Investigation, Formal analysis, Data curation, Conceptualization. CT: Writing – review & editing, Project administration, Funding acquisition.

## References

[1] World Health Organization. Breast cancer . Available online at: https://www.who.int/news-room/fact-sheets/detail/breast-cancer (Accessed 2024-01-03).

[2] Acevedo F Walbaum B Medina L Merino T Camus M Puschel K. Clinical characteristics, risk factors, and outcomes in Chilean triple negative breast cancer patients: a real-world study. Breast Cancer Res Treat. (2023) 197:449–59. doi: 10.1007/s10549-022-06814-x 36414796

[3] Ajabnoor GM. The molecular and genetic interactions between obesity and breast cancer risk. Medicina. (2023) 59:1338. doi: 10.3390/medicina59071338 37512149 PMC10384495

[4] Albain KS Gray RJ Makower DF Faghih A Hayes DF Geyer CE Jr.. Race, ethnicity, and clinical outcomes in hormone receptor-positive, her2-negative, node-negative breast cancer in the randomized tailorx trial. JNCI: J Natl Cancer Institute. (2021) 113:390–9. doi: 10.1093/jnci/djaa148 PMC8599918 32986828

[5] Alfian G Syafrudin M Fahrurrozi I Fitriyani NL Atmaji FTD Widodo T. Predicting breast cancer from risk factors using svm and extra-trees-based feature selection method. Computers. (2022) 11:136. doi: 10.3390/computers11090136

[6] Ali AS Nazar ME Mustafa RM Hussein S Qurbani K Ahmed SK. Impact of heavy metals on breast cancer. World Acad Sci J. (2024) 6:1–12. doi: 10.3892/wasj

[7] Almansour NM. Triple-negative breast cancer: a brief review about epidemiology, risk factors, signaling pathways, treatment and role of artificial intelligence. Front Mol Biosci. (2022) 9:836417. doi: 10.3389/fmolb.2022.836417 35145999 PMC8824427

[8] Alzu’bi A Najadat H Doulat W Al-Shari O Zhou L. Predicting the recurrence of breast cancer using machine learning algorithms. Multimedia Tools Appl. (2021) 80:13787–800. doi: 10.1007/s11042-020-10448-w

[9] Arafat HM Omar J Shafii N Naser IA Al Laham NA Muhamad R. The association between the serum level of igf-1 and igfbp-3 and the risk of breast cancer among women in the gaza strip. Asian Pacific J Cancer prevention: APJCP. (2023) 24:717. doi: 10.31557/APJCP.2023.24.2.717 PMC10162616 36853324

[10] Arceo-Martínez MT López-Meza JE Ochoa-Zarzosa A Palomera-Sanchez Z. Estado actual del cáncer de mama en méxico: principales tipos y factores de riesgo. Gaceta mexicana oncología. (2021) 20:101–10. doi: 10.24875/j.gamo.21000134

[11] Atakpa EC Buist DS Aiello Bowles EJ Cuzick J Brentnall AR. Development and evaluation of a method to assess breast cancer risk using a longitudinal history of mammographic density: a cohort study. Breast Cancer Res. (2023) 25:147. doi: 10.1186/s13058-023-01744-y 38001476 PMC10668455

[12] Azim HA Loutfy SA Azim HA Jr. Kamal NS Abdel Fattah NF Elberry MH. The landscape of brca mutations among Egyptian women with breast cancer. Oncol Ther. (2023) 11:445–59. doi: 10.1007/s40487-023-00240-9 PMC10673778 37731153

[13] Behravan H Hartikainen JM Tengström M Kosma VM Mannermaa A. Predicting breast cancer risk using interacting genetic and demographic factors and machine learning. Sci Rep. (2020) 10:11044. doi: 10.1038/s41598-020-66907-9 32632202 PMC7338351

[14] Bharati S Podder P Mondal M. Artificial neural network based breast cancer screening: a comprehensive review. arXiv preprint arXiv:2006.01767. (2020) 12:125–37. doi: 10.48550/arXiv.2006.01767

[15] Bhatt R van den Hout A Antoniou AC Shah M Ficorella L Steggall E. Estimation of age of onset and progression of breast cancer by absolute risk dependent on polygenic risk score and other risk factors. Cancer. (2024) 2–10. doi: 10.1002/cncr.35183 PMC7615824 38174903

[16] Bissan AD Ly M Amegonou AEH Sidibe FM Koné BS Barry NOK. Plasma 25-hydroxyvitamin d and 1, 25-dihydroxyvitamin d levels in breast cancer risk in Mali: A case–control study. Diagnostics. (2023) 13:3664. doi: 10.3390/diagnostics13243664 38132250 PMC10742900

[17] Botlagunta M Botlagunta MD Myneni MB Lakshmi D Nayyar A Gullapalli JS. Classification and diagnostic prediction of breast cancer metastasis on clinical data using machine learning algorithms. Sci Rep. (2023) 13:485. doi: 10.1038/s41598-023-27548-w 36627367 PMC9831019

[18] Bu R Siraj AK Al-Rasheed M Iqbal K Azam S Qadri Z. Identification and characterization of atm founder mutation in brca-negative breast cancer patients of arab ethnicity. Sci Rep. (2023) 13:20924. doi: 10.1038/s41598-023-48231-0 38017116 PMC10684510

[19] Campbell NJ Barton C Cutress RI Copson ER. Impact of obesity, lifestyle factors and health interventions on breast cancer survivors. Proc Nutr Soc. (2023) 82:47–57. doi: 10.1017/S0029665122002816 36426642

[20] Cao Y Xia B Zhang Z Hu D Huang X Yuan J. Association of body fat distribution and risk of breast cancer in pre-and postmenopausal women. Obes Facts. (2023) 16:356–63. doi: 10.1159/000529834 PMC10444009 36882014

[21] Castelló A Rodríguez-Barranco M Lope V Guevara M Colorado-Yohar S Dorronsoro A. High adherence to western dietary pattern increases breast cancer risk (an epic-Spain study). Maturitas. (2024) 179:107868. doi: 10.1016/j.maturitas.2023.107868 37925868

[22] Cavalcante LG Domingues RAR de Oliveira Junior B Fernandes MAR Pessoa EC Abbade LPF. Incidence of radiodermatitis and factors associated with its severity in women with breast cancer: a cohort study. Anais Brasileiros Dermatologia. (2024) 99:57–65. doi: 10.1016/j.abd.2023.01.004 PMC10964356 37657957

[23] Cheung BHH Man VCM Sham GTW Chow L Co M Kwong A. Pregnancy-related breast cancer: 14-year experience in a tertiary institution in hong kong. Cancer Treat Res Commun. (2024) 38:100783. doi: 10.1016/j.ctarc.2023.100783 38184967

[24] Chitkara A Mesa-Eguiagaray I Wild SH Hall PS Cameron DA Sims AH. Reproductive history differs by molecular subtypes of breast cancer among women aged 50 years in scotland diagnosed 2009–2016: a cross-sectional study. Breast Cancer Res Treat. (2022) 196:379–87. doi: 10.1007/s10549-022-06721-1 PMC9581868 36116093

[25] Christakoudi S Tsilidis KK Dossus L Rinaldi S Weiderpass E Antoniussen CS. A body shape index (absi) is associated inversely with post-menopausal progesterone-receptor-negative breast cancer risk in a large european cohort. BMC Cancer. (2023) 23:1–12. doi: 10.1186/s12885-023-11056-1 37337133 PMC10278318

[26] Clendenen TV Ge W Koenig KL Afanasyeva Y Agnoli C Bertone-Johnson E. Breast cancer risk factors and circulating anti-müllerian hormone concentration in healthy premenopausal women. J Clin Endocrinol Metab. (2021) 106:e4542–53. doi: 10.1210/clinem/dgab461 PMC8530718 34157104

[27] Crispo A Augustin LS Luongo A Calderaio C Breda J Coluccia S. Central obesity, body mass index, metabolic syndrome and mortality in mediterranean breast cancer patients. Sci Rep. (2023) 13:21208. doi: 10.1038/s41598-023-45439-y 38040773 PMC10692221

[28] Dammu H Ren T Duong TQ. Deep learning prediction of pathological complete response, residual cancer burden, and progression-free survival in breast cancer patients. PloS One. (2023) 18:e0280148. doi: 10.1371/journal.pone.0280148 36607982 PMC9821469

[29] Dehesh T Fadaghi S Seyedi M Abolhadi E Ilaghi M Shams P. The relation between obesity and breast cancer risk in women by considering menstruation status and geographical variations: A systematic review and meta-analysis. BMC Women’s Health. (2023) 23:392. doi: 10.1186/s12905-023-02543-5 37496015 PMC10373406

[30] DeRouen MC Yang J Li Y Franke AA Tome AN White KK. Circulating 27-hydroxycholesterol, lipids, and steroid hormones in breast cancer risk: a nested case–control study of the multiethnic cohort study. Breast Cancer Res. (2023) 25:95. doi: 10.1186/s13058-023-01693-6 37580793 PMC10424359

[31] Digkas E Smith DR Wennstig AK Matikas A Tegnelius E Valachis A. Incidence and risk factors of hypothyroidism after treatment for early breast cancer: a population-based cohort study. Breast Cancer Res Treat. (2023) 204:79–87. doi: 10.21203/rs.3.rs-3112497/v1 PMC10805818 38057688

[32] Duarte C Salazar A Strasser-Weippl K de Vries E Wiesner C Arango-Gutiérrez A. Breast cancer in Colombia: a growing challenge for the healthcare system. Breast Cancer Res Treat. (2021) 186:15–24. doi: 10.1007/s10549-020-06091-6 33611666

[33] Eren S Arslan A Çalişkan E Akay E Özhan N Topuz Ö. Comparison of clinical features and the impact of reproductive factors on by age at diagnosis young and elderly breast cancer patients in the middle anatolian region of Turkey. Eur Rev Med Pharmacol Sci. (2022) 26(7):2227–37. doi: 10.26355/eurrev_202204_28453 35442507

[34] Escala-Garcia M Morra A Canisius S Chang-Claude J Kar S Zheng W. Breast cancer risk factors and their effects on survival: a mendelian randomisation study. BMC Med. (2020) 18:1–10. doi: 10.1186/s12916-020-01797-2 33198768 PMC7670589

[35] Fakhri N Chad MA Lahkim M Houari A Dehbi H Belmouden A. Risk factors for breast cancer in women: an update review. Med Oncol. (2022) 39:197. doi: 10.1007/s12032-022-01804-x 36071255

[36] Fantin R Ulloa CS Solís CB. Social gradient in cancer incidence in Costa Rica: Findings from a national population-based cancer registry. Cancer Epidemiol. (2020) 68:101789. doi: 10.1016/j.canep.2020.101789 32795947

[37] Fei F Zhang K Siegal GP Wei S. A simplified breast cancer prognostic score: comparison with the ajcc clinical prognostic staging system. Modern Pathol. (2021) 34:2141–7. doi: 10.1038/s41379-021-00890-y 34365462

[38] Fentie H Ntenda PAM Tiruneh FN. Dietary pattern and other factors of breast cancer among women: a case control study in northwest Ethiopia. BMC Cancer. (2023) 23:1050. doi: 10.1186/s12885-023-11501-1 37915028 PMC10619250

[39] Giaquinto AN Sung H Miller KD Kramer JL Newman LA Minihan A. Breast cancer statistics, 2022. CA: Cancer J Clin. (2022) 72:524–41. doi: 10.3322/caac.21754 36190501

[40] Grigg J Manning V Cheetham A Greenwood CJ Youssef G Lockie D. Risky alcohol consumption among women in Australia attending breast screening services: an exploratory cross-sectional study. Addiction. (2023) 118:1493–506. doi: 10.1111/add.16191 37057463

[41] Guo Q Lu Y Liu W Lan G Lan T. The global, regional, and national disease burden of breast cancer attributable to tobacco from 1990 to 2019: a global burden of disease study. BMC Public Health. (2024) 24:107. doi: 10.1186/s12889-023-17405-w 38184557 PMC10770986

[42] Habel LA Alexeeff SE Achacoso N Arasu VA Gastounioti A Gerstley L. Examination of fully automated mammographic density measures using libra and breast cancer risk in a cohort of 21,000 non-hispanic white women. Breast Cancer Res. (2023) 25:92. doi: 10.1186/s13058-023-01685-6 37544983 PMC10405373

[43] Hathaway CA Rice MS Collins LC Chen D Frank DA Walker S. Prolactin levels and breast cancer risk by tumor expression of prolactin-related markers. Breast Cancer Res. (2023) 25:24. doi: 10.1186/s13058-023-01618-3 36882838 PMC9990334

[44] Herrán OF Álvarez DC Quintero-Lesmes DC. Dietary patterns and breast cancer in Colombia: an ecological study. Int Health. (2020) 12:317–24. doi: 10.1093/inthealth/ihz085 PMC7322202 31691807

[45] Hickman SE Baxter GC Gilbert FJ. Adoption of artificial intelligence in breast imaging: evaluation, ethical constraints and limitations. Br J Cancer. (2021) 125:15–22. doi: 10.1038/s41416-021-01333-w 33772149 PMC8257639

[46] Hindmarch S Howell SJ Usher-Smith JA Gorman L Evans DG French DP. Feasibility and acceptability of offering breast cancer risk assessment to general population women aged 30–39 years: a mixed-methods study protocol. BMJ Open. (2024) 14:e078555. doi: 10.1136/bmjopen-2023-078555 PMC10806663 38199637

[47] Hirko KA Rocque G Reasor E Taye A Daly A Cutress RI. The impact of race and ethnicity in breast cancer—disparities and implications for precision oncology. BMC Med. (2022) 20:1–12. doi: 10.1186/s12916-022-02260-0 35151316 PMC8841090

[48] Hook C Chatterjee U Sheng H Zhu Q Robinson T Roh JM. A polygenic score associated with fracture risk in breast cancer patients treated with aromatase inhibitors. NPJ Breast Cancer. (2024) 10:9. doi: 10.1038/s41523-024-00615-9 38245540 PMC10799916

[49] Huang MC Huang TT Feng HC Chen IC Chang CI Wang TN. Lifestyle factors and energy intakes with risks of breast cancer among pre-and post-menopausal women in Taiwan. Nutrients. (2023) 15:3900. doi: 10.3390/nu15183900 37764684 PMC10534793

[50] Islam MM Haque MR Iqbal H Hasan MM Hasan M Kabir MN. Breast cancer prediction: a comparative study using machine learning techniques. SN Comput Sci. (2020) 1:1–14. doi: 10.1007/s42979-020-00305-w

[51] Jiang R Wang X Sun Z Wu S Chen S Cai H. Association of education level with the risk of female breast cancer: a prospective cohort study. BMC Women’s Health. (2023) 23:1–6. doi: 10.1186/s12905-023-02245-y 36882777 PMC9993575

[52] Jin J Li J Liu Y Shi Q Zhang B Ji Y. Thyroid hormone changes correlate to combined breast cancer with primary thyroid cancer. Breast Cancer: Targets Ther. (2024) 16:15–22. doi: 10.2147/BCTT.S442707 PMC10787567 38223235

[53] Ju J Gao SL Wang JY Sang D Kang YK Wang X. Prognostic factors and benefit populations of ovarian function suppression in premenopausal hr+/her2+ early-stage breast cancer patients who received trastuzumab: evidence from a real-world study with long-term follow-up. Thorac Cancer. (2024) 15:439–47. doi: 10.1111/1759-7714.15211 PMC10883855 38185807

[54] Kamburova ZB Popovska SL Kovacheva KS Dimitrov DD Nikolova SE. Genetic predisposition in female patients with triple-negative breast cancer. World Acad Sci J. (2024) 6:1–8. doi: 10.3892/wasj.2023.217

[55] Kang E Jung JJ Lim C Kim HK Lee HB Han W. Increased risk of contralateral breast cancer for brca1/2 wild-type, high-risk korean breast cancer patients: a retrospective cohort study. Breast Cancer Res. (2024) 26:14. doi: 10.1186/s13058-024-01769-x 38254240 PMC10801954

[56] Kim G Bahl M. Assessing risk of breast cancer: a review of risk prediction models. J Breast Imaging. (2021) 3:144–55. doi: 10.1093/jbi/wbab001 PMC7980704 33778488

[57] Kim SH Jo HY. Factors associated with poor quality of life in breast cancer survivors: a 3-year follow-up study. Preprint. (2023) 15:2–12. doi: 10.20944/preprints202310.1934.v1 PMC10741455 38136354

[58] Kim N Kim H Park W Cho WK Kim TG Im YH. Impact of high dose radiotherapy for breast tumor in locoregionally uncontrolled stage iv breast cancer: a need for a risk-stratified approach. Radiat Oncol. (2023) 18:168. doi: 10.1186/s13014-023-02357-7 37821947 PMC10566115

[59] Kim HY Park J Moon SJ Jeong S Hong JH Lee JK. Short-term impact of hormone replacement therapy on risk of breast cancer in brca mutation carriers: A nationwide study in South Korea. Cancer Res Treat. (2024) 56:143. doi: 10.4143/crt.2023.653 37591780 PMC10789953

[60] Ko C Brody JP. Evaluation of a genetic risk score computed using human chromosomal-scale length variation to predict breast cancer. Hum Genomics. (2023) 17:1–8. doi: 10.1186/s40246-023-00482-8 37328908 PMC10273758

[61] Kyalwazi B Yau C Campbell MJ Yoshimatsu TF Chien AJ Wallace AM. Race, gene expression signatures, and clinical outcomes of patients with high-risk early breast cancer. JAMA Network Open. (2023) 6:e2349646–e2349646. doi: 10.1001/jamanetworkopen.2023.49646 38153734 PMC10755617

[62] Lalioti A Verzeletti L Tiberio P Gerosa R Gaudio M Saltalamacchia G. Common misconceptions about diet and breast cancer: An unclear issue to dispel. Cancers. (2024) 16:306. doi: 10.3390/cancers16020306 38254795 PMC10814151

[63] Li J Zhou Z Dong J Fu Y Li Y Luan Z. Predicting breast cancer 5-year survival using machine learning: A systematic review. PloS One. (2021) 16:e0250370. doi: 10.1371/journal.pone.0250370 33861809 PMC8051758

[64] Lima SM Kehm RD Swett K Gonsalves L Terry MB. Trends in parity and breast cancer incidence in us women younger than 40 years from 1935 to 2015. JAMA network Open. (2020) 3:e200929–e200929. doi: 10.1001/jamanetworkopen.2020.0929 32167569 PMC7070232

[65] Lima SM Kehm RD Terry MB. Global breast cancer incidence and mortality trends by region, age-groups, and fertility patterns. EClinicalMedicine. (2021) 38. doi: 10.1016/j.eclinm.2021.100985 PMC8271114 34278281

[66] Lin E Sleboda P Rimel BJ Datta GD. Inequities in colorectal and breast cancer screening: At the intersection of race/ethnicity, sexuality, and gender. SSM-Population Health. (2023) 24:101540. doi: 10.1016/j.ssmph.2023.101540 37920304 PMC10618777

[67] Lipsyc-Sharf M Ballman KV Campbell JD Muss HB Perez EA Shulman LN. Age, body mass index, tumor subtype, and racial and ethnic disparities in breast cancer survival. JAMA Network Open. (2023) 6:e2339584–e2339584. doi: 10.1001/jamanetworkopen.2023.39584 37878313 PMC10600583

[68] Liu R Ospanova S Perry RJ. The impact of variance in carnitine palmitoyltransferase-1 expression on breast cancer prognosis is stratified by clinical and anthropometric factors. PloS One. (2023) 18:e0281252. doi: 10.1371/journal.pone.0281252 36735704 PMC9897541

[69] Łukasiewicz S Czeczelewski M Forma A Baj J Sitarz R Stanisławek A. Breast cancer—epidemiology, risk factors, classification, prognostic markers, and current treatment strategies—an updated review. Cancers. (2021) 13:4287. doi: 10.3390/cancers13174287 34503097 PMC8428369

[70] Lv L Zhao B Kang J Li S Wu H. Trend of disease burden and risk factors of breast cancer in developing countries and territories, from 1990 to 2019: Results from the global burden of disease study 2019. Front Public Health. (2023) 10:1078191. doi: 10.3389/fpubh.2022.1078191 36726635 PMC9884979

[71] Ma L Liu A Gao J Zhao H. The prognostic impact of body mass index on female breast cancer patients in underdeveloped regions of northern China differs by menopause status and tumor molecular subtype. Open Life Sci. (2023) 18:20220748. doi: 10.1515/biol-2022-0748 37941781 PMC10628583

[72] Mao X Omeogu C Karanth S Joshi A Meernik C Wilson L. Association of reproductive risk factors and breast cancer molecular subtypes: a systematic review and meta-analysis. BMC Cancer. (2023) 23:644. doi: 10.1186/s12885-023-11049-0 37430191 PMC10334550

[73] Martinez RG van Dongen DM. Deep learning algorithms for the early detection of breast cancer: a comparative study with traditional machine learning. Inform Med Unlocked. (2023) 41:2–8. doi: 10.1016/j.imu.2023.101317

[74] Mazo C Aura C Rahman A Gallagher WM Mooney C. Application of artificial intelligence techniques to predict risk of recurrence of breast cancer: A systematic review. J Personalized Med. (2022) 12:1496. doi: 10.3390/jpm12091496 PMC9500690 36143281

[75] Mohammed AM Hamed HB Noaman MK Alieldin N. Metabolic syndrome and breast cancer risk. J Egyptian Natl Cancer Institute. (2023) 35:42. doi: 10.1186/s43046-023-00203-1 PMC13313949 38123741

[76] Mohanty SS Mohanty PK. Obesity as potential breast cancer risk factor for postmenopausal women. Genes Dis. (2021) 8:117–23. doi: 10.1016/j.gendis.2019.09.006 PMC8099684 33997158

[77] Morales-Pison S Tapia JC Morales-González S Maldonado E Acuña M Calaf GM. Association of germline variation in driver genes with breast cancer risk in Chilean population. Int J Mol Sci. (2023) 24:16076. doi: 10.3390/ijms242216076 38003265 PMC10671568

[78] Mousa NA Marino N Simões BM. Sex steroid hormones: effects on breast cancer risk and etiology. Front Endocrinol. (2023) 14:1198770. doi: 10.3389/fendo.2023.1198770 PMC10154670 37152952

[79] Mubtasim N Moustaid-Moussa N Gollahon L. The complex biology of the obesity-induced, metastasis-promoting tumor microenvironment in breast cancer. Int J Mol Sci. (2022) 23:2480. doi: 10.3390/ijms23052480 35269622 PMC8910079

[80] Nabila S Choi JY Abe SK Islam MR Rahman MS Saito E. Differential patterns of reproductive and lifestyle risk factors for breast cancer according to birth cohorts among women in China, Japan and korea. Breast Cancer Res. (2024) 26:15. doi: 10.1186/s13058-024-01766-0 38254178 PMC10801993

[81] Naji MA El Aarika K Benlahmar EH Abdelouhahid RA Debauche O. Machine learning algorithms for breast cancer prediction and diagnosis. Proc Comput Sci. (2021) 191:487–92. doi: 10.1016/j.procs.2021.07.062

[82] Nikolaienko O Eikesdal HP Ognedal E Gilje B Lundgren S Blix ES. Prenatal brca1 epimutations contribute significantly to triple-negative breast cancer development. Genome Med. (2023) 15:104. doi: 10.1186/s13073-023-01262-8 38053165 PMC10698991

[83] O’Neill B Yusuf A Lofters A Huang A Ekeleme N Kiran T. Breast cancer screening among females with and without schizophrenia. JAMA network Open. (2023) 6:e2345530–e2345530. doi: 10.1001/jamanetworkopen.2023.45530 38019514 PMC10687664

[84] Oza P. Chapter 2 - ai in breast imaging: Applications, challenges, and future research. In: Hemanth DJ, editor. Computational Intelligence and Modelling Techniques for Disease Detection in Mammogram Images. Academic Press (2024). p. 39–54.

[85] Pakzad R Nedjat S Salehiniya H Mansournia N Etminan M Nazemipour M. Effect of alcohol consumption on breast cancer: probabilistic bias analysis for adjustment of exposure misclassification bias and confounders. BMC Med Res Method. (2023) 23:157. doi: 10.1186/s12874-023-01978-6 PMC10318777 37403100

[86] Pearson SA Taylor S Marsden A O’Reilly JD Krishan A Howell S. Geographic and sociodemographic access to systemic anticancer therapies for secondary breast cancer: a systematic review. Systematic Rev. (2024) 13:35. doi: 10.1186/s13643-023-02382-3 PMC10795363 38238821

[87] Pedersini R Laganà M Bosio S Zanini B Cosentini D di Mauro P. Is weight gain preventable in women with early breast cancer undergoing chemotherapy? a real-world study on dietary pattern, physical activity, and body weight before and after chemotherapy. Breast Cancer Res Treat. (2023) 202:461–71. doi: 10.1007/s10549-023-07095-8 PMC10564810 37695400

[88] Pfob A Mehrara BJ Nelson JA Wilkins EG Pusic AL Sidey-Gibbons C. Machine learning to predict individual patient-reported outcomes at 2-year follow-up for women undergoing cancer-related mastectomy and breast reconstruction (inspired-001). Breast. (2021) 60:111–22. doi: 10.1016/j.breast.2021.09.009 PMC8551470 34619573

[89] Roheel A Khan A Anwar F Akbar Z Akhtar MF Khan MI. Global epidemiology of breast cancer based on risk factors: a systematic review. Front Oncol. (2023) 13:1–15. doi: 10.3389/fonc.2023.1240098 PMC10598331 37886170

[90] Schonberg MA Wolfson EA Eliassen AH Bertrand KA Shvetsov YB Rosner BA. A model for predicting both breast cancer risk and non-breast cancer death among women¿ 55 years old. Breast Cancer Res. (2023) 25:8. doi: 10.1186/s13058-023-01605-8 36694222 PMC9872276

[91] Schreurs MA Ramón Y Cajal T Adank MA Collée JM Hollestelle A van Rooij J. The benefit of adding polygenic risk scores, lifestyle factors, and breast density to family history and genetic status for breast cancer risk and surveillance classification of unaffected women from germline chek2 c. 1100delc families. Breast. (2024) 73:103611. doi: 10.1016/j.breast.2023.103611 38039887 PMC10730863

[92] Schwartz CJ Khorsandi N Blanco A Mukhtar RA Chen YY Krings G. Clinicopathologic and genetic analysis of invasive breast carcinomas in women with germline chek2 variants. Breast Cancer Res Treat. (2023) 204:171–9. doi: 10.1007/s10549-023-07176-8 PMC10806021 38091153

[93] Shi J Liu T Ge Y Liu C Zhang Q Xie H. Cholesterol-modified prognostic nutritional index (cpni) as an effective tool for assessing the nutrition status and predicting survival in patients with breast cancer. BMC Med. (2023) 21:512. doi: 10.1186/s12916-023-03225-7 38129842 PMC10740286

[94] Silva RJG Grippa WR Neto LCBS Enriquez-Martinez OG Marcarini JAC Pessanha RM. Factors associated with the nutritional status of women with non-metastatic breast cancer in a Brazilian high complexity oncology center. Nutrients. (2023) 15:4961. doi: 10.3390/nu15234961 38068818 PMC10707825

[95] Silva-Aravena F Núñez Delafuente H Gutiérrez-Bahamondes JH Morales J. A hybrid algorithm of ml and xai to prevent breast cancer: a strategy to support decision making. Cancers. (2023) 15:2443. doi: 10.3390/cancers15092443 37173910 PMC10177162

[96] Smotherman C Sprague B Datta S Braithwaite D Qin H Yaghjyan L. Association of air pollution with postmenopausal breast cancer risk in uk biobank. Breast Cancer Res. (2023) 25:83. doi: 10.1186/s13058-023-01681-w 37443054 PMC10339564

[97] Speiser D Bick U. Primary prevention and early detection of hereditary breast cancer. Breast Care. (2023) 18:448–54. doi: 10.1159/000533391 PMC10730103 38125920

[98] Sun X Reiner AS Tran AP Watt GP Oh JH Mellemkjær L. A genome-wide association study of contralateral breast cancer in the women’s environmental cancer and radiation epidemiology study. Breast Cancer Res. (2024) 26:16. doi: 10.1186/s13058-024-01765-1 38263039 PMC10807183

[99] Tarighati E Keivan H. Mahani H. A review of prognostic and predictive biomarkers in breast cancer. Clin Exp Med. (2023) 23:1–16. doi: 10.1007/s10238-021-00781-1 35031885

[100] Torres-Roman JS Martinez-Herrera JF Carioli G Ybaseta-Medina J Valcarcel B Pinto JA. Breast cancer mortality trends in Peruvian women. BMC Cancer. (2020) 20:1–9. doi: 10.1186/s12885-020-07671-x PMC7706041 33261561

[101] Tüchler A De Pauw A Ernst C Anota A Lakeman IM Dick J. Clinical implications of incorporating genetic and non-genetic risk factors in canrisk-based breast cancer risk prediction. Breast. (2024) 73:103615. doi: 10.1016/j.breast.2023.103615 38061307 PMC10749276

[102] Tzenios N. Obesity as a risk factor for cancer. EPRA Int J Res Dev (IJRD). (2023) 8:101–4. doi: 10.36713/epra2013

[103] Vagnini D Natalucci V Moi S Vallorani L Pietrelli A Panico AR. Home-based lifestyle intervention for breast cancer survivors: A surprising improvement in the quality of life during the first year of covid-19 pandemic. PloS One. (2024) 19:e0296163. doi: 10.1371/journal.pone.0296163 38165970 PMC10760703

[104] Wang H MacInnis RJ Li S. Family history and breast cancer risk for asian women: a systematic review and meta-analysis. BMC Med. (2023) 21:239. doi: 10.1186/s12916-023-02950-3 37400822 PMC10318753

[105] Wekking D Lambertini M Dessì M Denaro N Bardanzellu F Garrone O. Cdk4/6 inhibitors in the treatment of metastatic breast cancer: focus on toxicity and safety. Semin Oncol (Elsevier). (2024) 17:2–8. doi: 10.1053/j.seminoncol.2024.01.002 38245458

[106] Wong LY Kapula N He H Guenthart BA Vitzthum LK Horst K. Risk of developing subsequent primary lung cancer after receiving radiation for breast cancer. JTCVS Open. (2023) 16:919–28. doi: 10.1016/j.xjon.2023.10.031 PMC10775166 38204675

[107] Xie Y Lei C Ma Y Li Y Yang M Zhang Y. Prognostic nomograms for breast cancer with lung metastasis: a seer-based population study. BMC Women’s Health. (2024) 24:16. doi: 10.1186/s12905-023-02848-5 38172874 PMC10765699

[108] Ye Z Nguyen TL Dite GS MacInnis RJ Schmidt DF Makalic E. Causal relationships between breast cancer risk factors based on mammographic features. Breast Cancer Res. (2023) 25:127. doi: 10.1186/s13058-023-01733-1 37880807 PMC10598934

[109] Ye L Hou Y Hu W Wang H Yang R Zhang Q. Repressed blautia-acetate immunological axis underlies breast cancer progression promoted by chronic stress. Nat Commun. (2023) 14:6160. doi: 10.1038/s41467-023-41817-2 37789028 PMC10547687

[110] Yogendran L Meis L Burnside E Schrager S. Management of women at high risk for breast cancer. J Am Board Family Med. (2024) 36:1029–32. doi: 10.3122/jabfm.2023.230064R1 37857439

[111] Yokokawa T Suzuki K Tsuji D Hosonaga M Kobayashi K Kawakami K. Influence of menopause on chemotherapy-induced nausea and vomiting in highly emetogenic chemotherapy for breast cancer: A retrospective observational study. Cancer Med. (2023) 12:18745–54. doi: 10.1002/cam4.6494 PMC10557896 37676079

[112] Zheng Y Li J Wu Z Li H Cao M Li N. Risk prediction models for breast cancer: a systematic review. BMJ Open. (2022) 12:e055398. doi: 10.1136/bmjopen-2021-055398

[113] Zhou M Henricks M Loch V Zhang G Lu Y Li X. Mendelian randomization analysis revealed potential metabolic causal factors for breast cancer. Sci Rep. (2023) 13:14290. doi: 10.1038/s41598-023-41130-4 37652957 PMC10471756

[114] Aria M Cuccurullo C. bibliometrix: an r-tool for comprehensive science mapping analysis. J Informetrics. (2017) 11:959–75. doi: 10.1016/j.joi.2017.08.007

