# Supplementary figures and images for: A contemporary review of breast cancer risk factors and the role of artificial intelligence
Source: Front Oncol. 2024 Apr 18;14:1356014. doi: 10.3389/fonc.2024.1356014 (PMC11063273; doi:10.3389/fonc.2024.1356014)

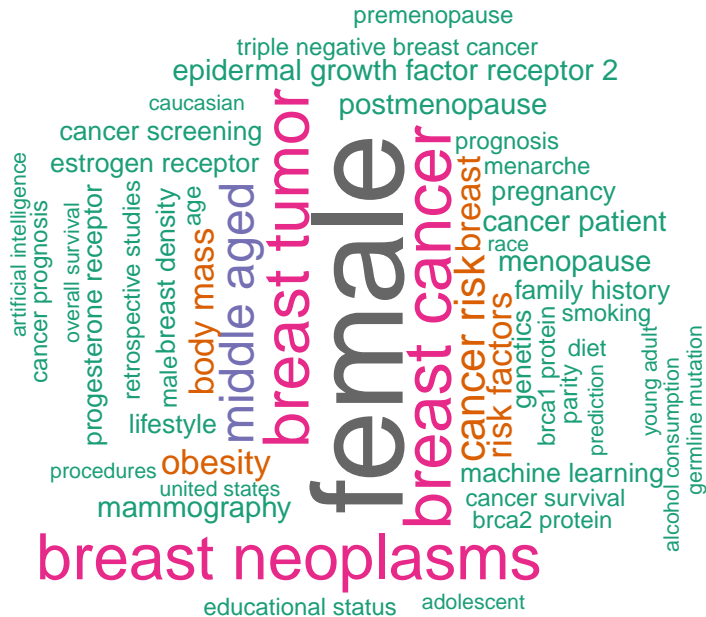

Supplement: Supplementary file 1 [file DataSheet_1.pdf]
